# Repatriation Data: More than two million species occurrence records added to the Brazilian Biodiversity Information Facility Repository (SiBBr)

**DOI:** 10.3897/BDJ.5.e12012

**Published:** 2017-05-30

**Authors:** David Dias, Clara Baringo Fonseca, Luiza Correa, Nayara Soto, Andrea Portela, Keila Juarez, Roque João Tumolo Neto, Murilo Ferro, João Gonçalves, Jurandir Junior

**Affiliations:** 1 Brazilian Biodiversity Information Facility (SiBBr), Brasília, Brazil

**Keywords:** Brazilian System for information on Biodiversity (SiBBr), primary biodiversity data, Global Biodiversity Information Facility (GBIF), repatriation data, occurrence records, Brazil

## Abstract

**Background:**

Primary biodiversity data records, available on-line, are essential for conservation planning. Of the mega diversity countries, Brazil have reached a high level of scientific research in describing their biodiversity. However, there still remain significant limitations in recovering, collating and organizing available information on Brazil's biological diversity and its distribution. Since the colonial period, biological material were often collected and transferred to other countries, which were characterized, stored and maintained. As a result, natural history museums worldwide possess large amounts of primary biodiversity data originally from Brazil which are then published on-line in the international Global Biodiversity Information Facility (GBIF) infrastructure. Aiming to recover these data, the Brazilian Biodiversity Information System (SiBBr) developed an automatic repatriation tool capable of retrieving all records registered in Brazil but published outside Brazilian territory in an automated manner.

**New information:**

Thus, 2,459,366 records were added to SiBBr’s Repository in one day. Europe and the United States hold about 80% of all records. The data set covers all life kingdoms. Animalia is the most represented group with 3 main phylum's: Chordata, Arthropoda and Mollusca, within more than 40% of all records. Plantae also comprises a large portion of the records with angiosperms having the major number of entries.

## Introduction

Biodiversity primary data are key to address scientific conservation and sustainability issues (Hardisty et al. 2013). Among several methods to disseminate biodiversity data, initiatives mainly using the Internet have become a challenge and a priority. Data should be available, discoverable and freely reusable worldwide. The Global Biodiversity Information Facility (GBIF) provides an international open data infrastructure that allows access to biodiversity data, including data from natural history collections (Wheeler 2004). Countries are encouraged to digitize their data and share it through the platform (Berendsohn et al. 2010) providing access to more than 700 million occurrence records from more than 880 publishers.

Brazil is classified at the top of the world’s 17 megadiverse countries, and second in terms of species endemism (Ginsberg 1999). It hosts between 15-20% of the world’s biological diversity with new species reported each year. Although Brazil have achieved a high level of scientific research, with an extensive system of academic and research instituitions (Scarano 2007) there still remain significant limitations in recovering, collating and organising available information on Brazil's biological diversity and its distribution. Historically, Brazil has raised interest for its natural resources and biodiversity since colonial times. According to Leite 1995firstly it was limited to citizens of Portugal who were instructed to discover natural resources and their uses. Later on, during 18th and 19th century, other regions of Europe concerned with observing and classifying natural specimens organized scientific expeditions to Brazil. Referred as Naturalists and sponsored by noblemen or scientific societies, biologists and other researchers travelled around Brazil with the purpose of discovering flora and fauna. Therefore, biological material was often collected and transferred to other countries, which were characterized, stored and maintained. As a result, natural history museums worldwide possess huge collections of Brazilian biodiversity that are not easily accessible to researchers in the countries from which they were collected (Santos 2016, Edwards 2004). Part of these data have been digitalized and nowadays are available on GBIF.

Due to the importance of making such data available to the countries of origin, the Convention Biological Diversity (CBD) and GBIF have called for the increased mutual transfer of biodiversity data between countries, also referred to as the repatriation process (Laihonen et al. 2004). Repatriation contributes significantly to the scientific and technological development of the country, preserving its biological diversity and genetic heritage. Both must be safeguarded because of its ecological value as an integral element of the environment and the foundation of socioeconomic activities. Furthermore, repatriation allows biodiversity information data to be transferred and published in national collections, museums and on-line repositories, such as the Brazilian Biodiversity Information Facility (SiBBr). Since 2011, SiBBr represents Brazil in GBIF, offering infrastructure that stimulates and facilitates the publication, integration, access and use of information about Brazilian biodiversity to the community. SiBBr currently integrates more than 10 million records from biological collections of Brazil.

Aiming to repatriate digital data from other countries, the SiBBr developed an automatic repatriation tool capable of retrieving all GBIF records within Brazilian coordinates published outside Brazilian territory and indexing them in the SiBBr repository as a dataset that is periodically updated. The present data paper describes the repatriation data set published in SiBBr’s repository through the Integration Publishing Toolkit (IPT) and list the steps of the automated repatriation process.

## Project description

### Title

Brazilian Biodiversity Information Facility (SiBBr)

### Design description

**Figure 1.**
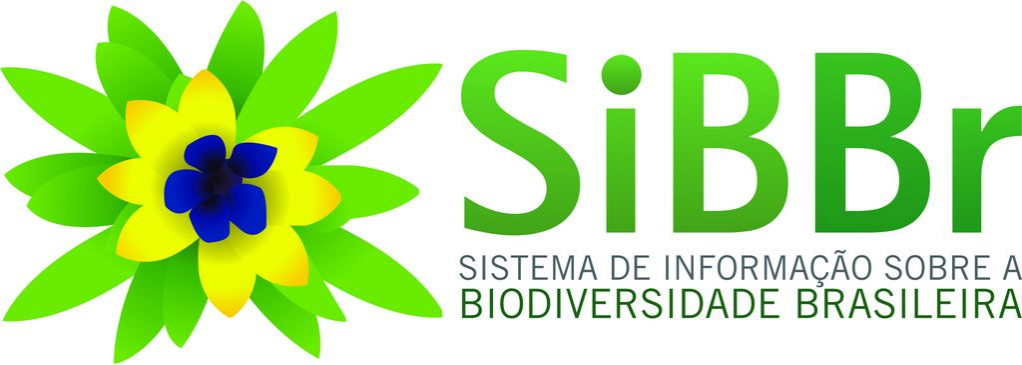
The Brazilian Biodiversity Information Facility - www.sibbr.gov.br

The Brazilian Biodiversity Information Facility, known as SiBBr (Fig. 1) is the national-wide system for biodiversity data. The project concept came as request from the Ministry of Science, Technology, Innovations and Communications of Brazil (MCTiC) due to the lack of an infrastructure to organize and assemble biodiversity information. Implemented in partnership with The United Nations Environment Program (UNEP) and funded by the Global Environment Facility (GEF), SiBBr represents the Brazilian web portal to make biodiversity databases available at a national level and worldwide through GBIF.

The SiBBr project goal is to ensure data-driven policy design and implementation by facilitating and mainstreaming biodiversity information into decision-making and policy development processes. Biodiversity primary data should be available to support strategic environmental action plans and official documents used by government agencies to identify priority areas for conservation, as well as procedures in the area of environmental licensing and impacts on biodiversity. The implementation is based on a collaborative network of institutions and actors where investments focus on the digitalization and modernization of biological collections and information to incorporate and use through the national on-line SiBBr repository.

SiBBr also provides instruments, tools and technology to support scientific research to expand base knowledge and the current capacity of learning about Brazilian biodiversity. The production of scientific knowledge will contribute the requirements of the society and allow decision-makers to establish policies that integrate biodiversity conservation and sustainable use objectives. SiBBr currently integrates approximately 300 datasets from 93 publishers between national and private institutions sharing more than 10 million records, including the repatriation data set.

## Sampling methods

### Sampling description

Data published in GBIF provide quick and easy access to global biodiversity data. Data users can search for specific data by customizing the search using filters such as publishing country or country of record which allows to find any data type. This procedure, done manually and on-line, is prolonged and a time-consuming effort. To avoid the procedure and aiming to speed up the process, in Brazil, repatriation of data from GBIF is automatic and periodic. The SiBBr team developed a tool that performs such action in an automated fashion indexing data in the SiBBr repository as it is placed in the system. Developed with Golang programming (https://golang.org/) and bash scripting, the source code comprises two different filters; country of origin (Brazil) and publishing country.

First of all, the repatriation tool makes an API request in GBIF database. Consequently, GBIF compiles all records that meets the conditions previously determined and retrieves a Comma Separated Values (csv) zipped file. Then, the csv file is converted to a sqlite database and published again through GBIF's Integrated Publishing Toolkit (Robertson et al. 2014) hosted in SiBBr.

However, data quality arrangements must be done before publish it again in SiBBr's repository through IPT. The tool is an open software developed to facilitate the share and usability of biodiversity primary data using a vocabulary or set of terms, named as Darwin Core (http://rs.tdwg.org/dwc/terms/) that describe biodiversity data (Berendsohn et al. 2010). Data from GBIF comes with restrictions based on modifications of the requirements for publication in IPT. In the current IPT version some fields are mandatory such the Darwin Core terms BasisOfRecord and occurrenceID. The term Basis of Record (the specific nature of the data record) uses a controlled vocabulary: "PreservedSpecimen", "FossilSpecimen", "LivingSpecimen", "HumanObservation", "MachineObservation". However, old versions of the IPT had a different controlled vocabulary. Instead of Human observation it was "observation" or "literature". Therefore, some modifications were made to adjust this requirement to publish in IPT. Finally, occurrenceID was rewritten to avoid duplicity.

The data paper describes the state of the data set when the procedure was used to harvest from GBIF for the first time on 9th of April of 2016, at which time 2,459,366 records were added into the SiBBr repository.

## Geographic coverage

### Description

**Figure 2.**
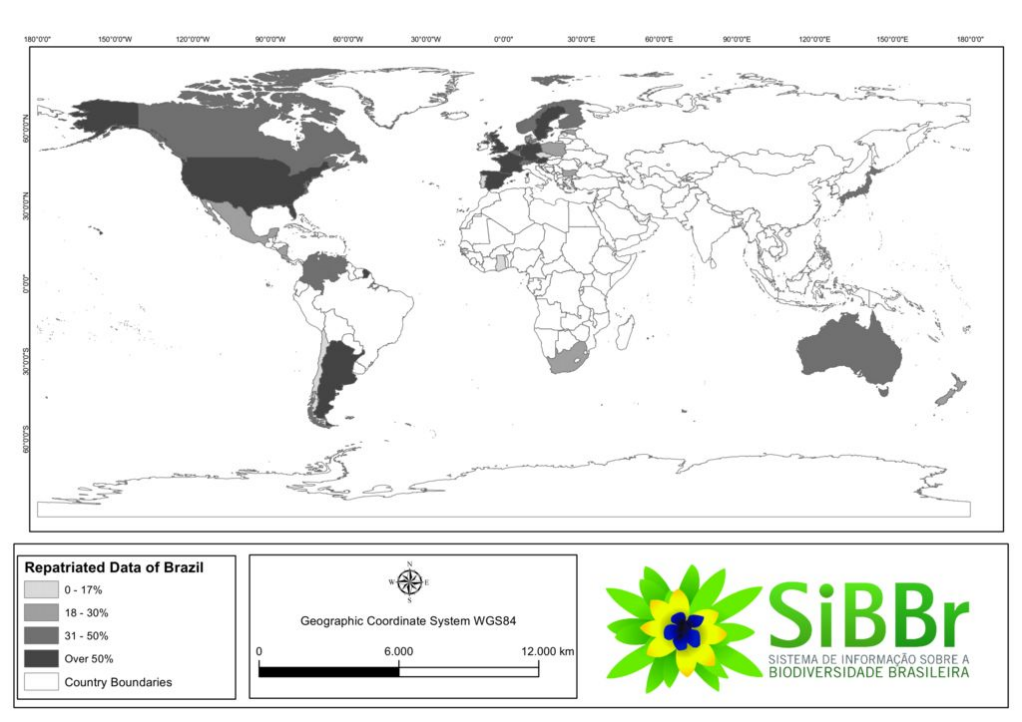
Geographic coverage by publishing country of the repatriation data set.

**Figure 3.**
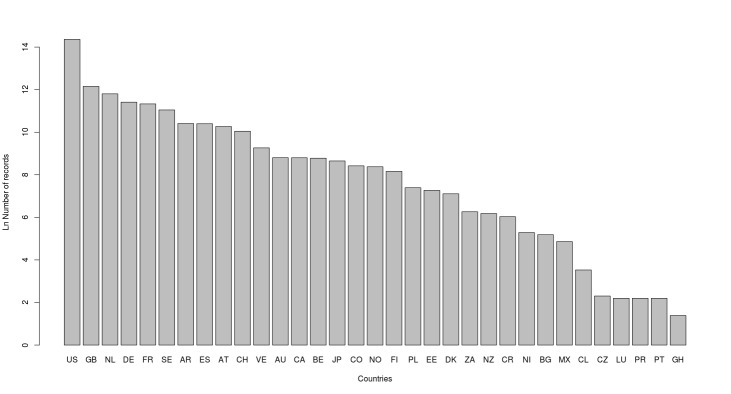
Number of Brazilian records per country published outside national borders (Logarithmic scale). US = United States of America; GB = United Kingdom; NL = Netherlands; DE = Germany; FR = France; SE = Sweden; AR = Argentina; ES = Spain; AT = Austria; CH = Switzerland; VE = Venezuela; AU = Australia; CA = Canada; BE = Belgium; JP = Japan; CO = Colombia; NO = Norway; FI = Finland; PL = Poland; EE = Estonia; DK = Denmark; ZA = South Africa; NZ = New Zealand; CR = Costa Rica; NI = Nicaragua; BG = Bulgaria; MX= Mexico; CL = Chile; CZ = Czech Republic; LU = Luxembourg; PR = Puerto Rico; PT = Portugal; GH = Ghana

**Figure 4.**
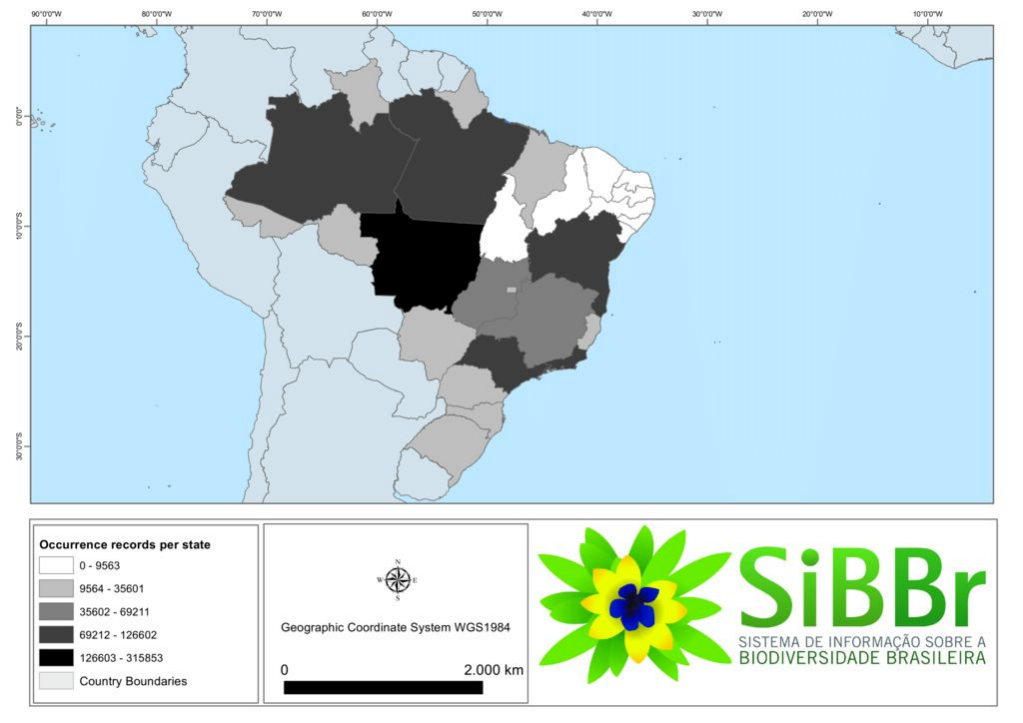
Geographic coverage by records of occurrence by Brazilian state of the repatriated data set

A total of 2,459,366 records have been distributed among all publishing countries worldwide. Figs 2, 3 give a representation of publishing countries with a major number of Brazilian occurrence records. The United States and Great Britain followed by the Netherlands, Denmark and other European countries and Argentina published the majority of all repatriated records. The most significant amount of data was collected in the Brazilian state of Mato Grosso, followed by Pará and Amazonas state (Fig. 4).

## Taxonomic coverage

### Description

**Figure 5.**
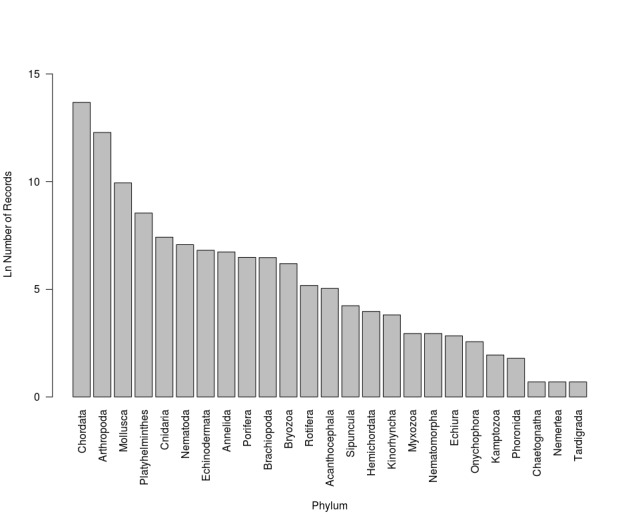
Taxonomic coverage by phylum among kingdom Animalia (Logarithmic scale).

The repatriation dataset comprises 2.459.366 records of all six life kingdoms; Animalia, Plantae, Fungi, Bacteria, Protozoa and Chromista. The best represented kingdom is Animalia with 25 phyla; Chordata, Arthropoda, Mollusca and Platyhelminthes have the most records. Other pylums include Cnidaria, Nematoda., Echinodermata, Annelida, Porifera, Brachiopoda, Bryozoa, Rotifera, Acanthocephala, Sipuncula, Hemichordata, Kinorhyncha, Myxozoa, Nematomorpha, Echiura, Onychophora, Kamptozoa, Phoronida, Chaetognatha, Chaetognatha, Nemertea and Tardigrada (Fig. 5).

**Figure 6.**
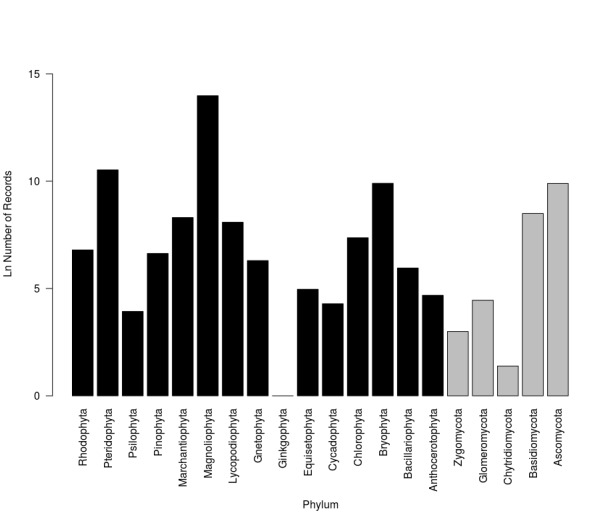
Number of occurrence records distributed among Plantae (black bars) and Fungi (gray bars) kingdoms by phyla published outside national borders (Logarithmic scale).

For Plantae, as despicted in Fig. 6 majority of records belong to phylum Magnoliophyta and Pteridophyta, followed by Bryophyta, Marchantiophyta and Lycopodiophyta. Other groups represented are Psilophyta, Gnetophyta, Ginkgophyta, Equisetophyta, Cycadophyta, Anthocerotophyta and three groups of algae; Chlorophyta, Rhodophyta and Bacillariophyta.

**Figure 7.**
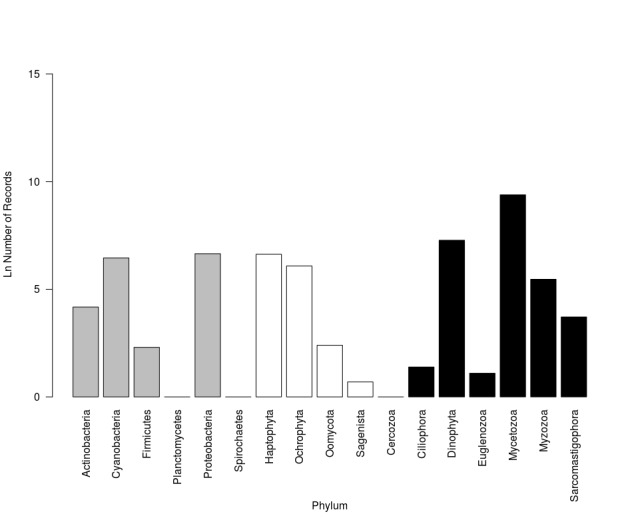
Number of occurrence records distributed among Chromista (white bars), Bacteria (gray bars) and Protozoa (black bars) kingdoms published outside national borders (logarithmic scale).

Regarding Fungi, the dataset includes 5 groups: Ascomycota, Basidiomycota, Glomeromycota, Zygomycota and Chytridiomycota (Fig. 6). Finally, there are 4 groups of Chroomista: Haptophyta, Ochrophyta, Oomycota and Sagenista and five phyla of Protozoa: Cercozoa, Ciliophora, Dinophyta, Euglenozoa, Mycetozoa, Myzozoa and Sarcomastigophora (Fig. 7).

## Temporal coverage

### Notes

All data repatriated comprise a collecting period of time that goes from 1658 to 2016. The first record available in GBIF from Brazil is based on a specimen collected in July of 1658. The specimen belong to phylum Spermatophyta, kingdom Plantae published in GBIF by The United States and stored in The Field Museum of Natural History of Chicago.

## Usage rights

### Use license

Creative Commons Public Domain Waiver (CC-Zero)

## Data resources

### Data package title

Repatriados

### Resource link

http://ipt.sibbr.gov.br/repatriados/resource?r=repatriados

### Number of data sets

1

### Data set 1.

#### Data set name

Dados Repatriados

#### Data format

Darwin Core Archive .dwca

#### Number of columns

1

#### Character set

UTF - 8

#### Download URL

http://ipt.sibbr.gov.br/repatriados/archive.do?r=repatriados

#### Data format version

1.0

#### 

**Data set 1.** 

Column label	Column description
Registro	Id of each single record
